# Effects of lithium on suicide and suicidal behaviour: a systematic review and meta-analysis of randomised trials

**DOI:** 10.1017/S204579602200049X

**Published:** 2022-09-16

**Authors:** Zainab Nabi, Jacki Stansfeld, Martin Plöderl, Lisa Wood, Joanna Moncrieff

**Affiliations:** 1 Division of Psychiatry, University College London, London, GB; 2 Department for Clinical Psychology and Department for Inpatient Psychotherapy and Crisis Intervention, University Clinic for Psychiatry, Psychotherapy and Psychosomatics, Paracelsus Medical University, Salzburg, Austria

**Keywords:** Bipolar disorder, depression, lithium, mood disorders unipolar, suicide

## Abstract

**Aims:**

Lithium has long been believed to reduce the risk of suicide and suicidal behaviour in people with mood disorders. Previous meta-analyses appeared to support this belief, but excluded relevant data due to the difficulty of conducting meta-analysis of rare events. The current study is an updated systematic review and meta-analysis that includes all eligible data, and evaluates suicide, non-fatal suicidal behaviour (including suicidal ideation) and suicide attempts.

**Methods:**

We searched PubMed, PsycINFO and Embase and some trial registers. We included all randomised trials comparing lithium and placebo or treatment as usual in mood disorders published after 2000, to ensure suicide was reliably reported. Trial quality was assessed using the Cochrane Risk of Bias tool. Pooled data were analysed using Fisher's Exact test. In addition, meta-analysis was conducted using various methods, prioritizing the Exact method. All trials were included in the analysis of suicide initially, regardless of whether they reported on suicide or not. We conducted a sensitivity analysis with trials that specifically reported on suicides and one that included trials published before 2000. Pre-specified subgroup analyses were performed involving suicide prevention trials, trials excluding people already taking lithium, trials involving people with bipolar disorder exclusively and those involving people with mixed affective diagnoses. Non-fatal suicidal behaviour and suicide attempts were analysed using the same methods, but only trials that reported these outcomes were included. PROSPERO registration: CRD42021265809.

**Results:**

Twelve eligible studies involving 2578 participants were included. The pooled suicide rate was 0.2% for people randomised to lithium and 0.4% with placebo or treatment as usual, which was not a statistically significant difference; odds ratio (OR) = 0.41 (95% confidence interval 0.03–2.49), *p* = 0.45. Meta-analysis using the Exact method produced an OR of 0.42 (95% confidence interval 0.01–4.5). The result was not substantially different when restricted to 11 trials that explicitly reported suicides and remained statistically non-significant when including 15 trials published before 2000 (mostly in the 1970s). There were no significant differences in any subgroup analysis. There was no difference in rates of all non-fatal suicidal behaviour in seven trials that reported this outcome, or in five trials that reported suicide attempts specifically. Meta-analyses using other methods also revealed no statistically significant differences.

**Conclusions:**

Evidence from randomised trials is inconclusive and does not support the idea that lithium prevents suicide or suicidal behaviour.

## Introduction

Suicide is a leading cause of death all over the world, accounting for 1.3% of all global deaths in 2019 (World Health Organisation, 2021a). In the USA, suicide was the 10th leading cause of death in 2019, and the second leading cause in young people (National Institute of Mental Health, 2022), although 77% of suicides occur in low- or middle-income countries (World Health Organisation, 2021b). Suicide attempts are at least 20 times as common as suicide (World Health Organisation, 2022), and are especially frequent among young people (O'Connor *et al*., 2018; Sivertsen *et al*., 2019). They cause substantial service use and economic costs (Royal College of Psychiatrists, 2010; Hawton *et al*., 2013) and are also a strong predictor of subsequent suicide (Chan *et al*., 2016).

Mood disorders increase the risk of suicide and suicide attempts. Long-term data suggest around 3% of people hospitalised with a diagnosis of bipolar disorder subsequently die by suicide, and 1.5% of those diagnosed with unipolar depression (Nordentoft *et al*., 2011). Suicide attempts and self-harm are also more common in people with mood disorders (Weintraub *et al*., 2017).

Lithium was first suggested to have anti-suicidal properties by Mogens Schou in 1954 (Schou *et al*., 1954), and the claim was repeated in the 1970s in a well-known report on the links between suicide and mental disorder (Barraclough, 1972). More recently, several leading researchers have supported the idea that lithium can prevent suicide, based initially on cohort studies (Baldessarini *et al*., 2006). An influential meta-analysis of randomised trials also concluded that lithium can reduce the risk of suicide (Cipriani *et al*., 2013). Moreover, ecological analyses have reported associations between lithium levels in drinking water and lower suicide rates (Memon *et al*., 2020), although publication bias and heterogeneity are noted as limitations in the latest meta-analysis of these studies (Eyre-Watt *et al*., 2021). Thus, despite some recent analyses coming to different conclusions (Riblet *et al*., 2017; Borjesson and Gotzsche, 2019), many researchers and clinicians regard lithium's anti-suicidal properties as ‘proven’ (Lewitzka *et al*., 2015a, p. 1) by ‘unambiguous evidence’ (Lewitzka *et al*., 2015b, p. 1). Lithium has been suggested to have an ‘intrinsic anti-suicidal property’ (Del Matto *et al*., 2020), and is recommended in some clinical practice guidelines for the prevention of suicidal behaviour (Veterans Affairs and Department of Defence, (2019). There have been calls to make guideline recommendations more assertive (Smith and Cipriani, 2017) and for lithium to be added to drinking water (Daly, 2020).

Drawing conclusions about a rare event such as suicide is always difficult, however, and previous studies suffer from methodological limitations. Most importantly, all meta-analyses conducted to date used the Peto method, which excludes studies in which no outcome events occur, and has recently been suggested to be problematic for this reason (Ren *et al*., 2019). Since suicide is so rare, many trials with relevant data have not been included in these analyses, which may have inflated treatment effects. We set out to conduct a meta-analysis of the effects of lithium on suicide and non-fatal suicidal behaviour using data from all eligible trials.

## Methods

This systematic review was conducted following guidance from the Preferred Reporting Items for Systematic Reviews and Meta-Analyses (PRISMA) guidelines for best-practice standards (Page *et al*., 2021). The protocol was developed and registered prior to data extraction (PROSPERO ID: CRD42021265809; https://www.crd.york.ac.uk/prospero/display_record.php?RecordID = 265809).

### Search strategy

PubMed, PsycINFO, Embase, ClinicalTrial.gov and the Cochrane Schizophrenia Group trial register were searched using the following terms: [‘lithium’] AND [‘affective disorder*’ OR ‘mood disorder*’ OR ‘depress*’ OR ‘bipolar’ OR ‘schizoaffective’ OR ‘personality disorder’ OR ‘dysthymia’ OR ‘rapid cycling’] AND [‘randomised control trial’ OR ‘RCT’ OR ‘trial’ OR ‘random*’]. In addition to terms for bipolar disorder and depression, additional mood diagnostic categories were included to ensure no relevant papers were missed (see online Supplementary Table S1). Searches were conducted up until March 2022. Google Scholar was searched to identify previous reviews and meta-analyses of similar topics, and a hand-search of the reference lists allowed for further studies to be identified. For protocols or ongoing trials, the researchers were contacted to determine if the studies had been completed and were near publication.

### Study selection

Inclusion criteria consisted of randomised control trials published in peer-reviewed literature that compared lithium with placebo or treatment as usual in the medium and long-term (>12 weeks) treatment of mood disorders. We included studies that employed treatment as usual, in which participants were not blinded, since we suspected that even when a placebo is used, blinding is likely to be inadequate due to the noticeable side effects of lithium. Participants were required to be over the age of 18 years and to have a diagnosis of a mood disorder made clinically or according to standardised diagnostic criteria manuals. Studies needed to be published after the year 2000 due to more modern reporting requirements introduced by the CONSORT statement (Begg *et al*., 1996) ensuring completeness of reporting. In older trials, suicide may not always have been reported, and since suicide is so rare, even one or two unreported events would have a substantial impact on the analysis. No language restrictions were applied.

Trials conducted to evaluate relapse prevention in mood disorders and trials specifically aimed at prevention of suicide and suicidal behaviour were eligible for inclusion. Trials in which lithium was used alone or in conjunction with other treatments were included where other treatments were available to the control group too. Comparative studies comparing lithium with another active drug that did not include a placebo or treatment as usual group were excluded.

### Study selection

All titles and abstracts identified by the search were screened by ZN and independently checked by JM. Full-text screening of the remaining studies was conducted by ZN and a sample was double checked by JM. Any uncertainties relating to inclusion were discussed and resolved with JM and JS.

### Quality assessment

The risk of bias in the included studies was assessed using an operationalised version of the original Cochrane Risk of Bias tool (Higgins and Green, 2011) for consistency with a previous influential meta-analysis (Cipriani *et al*., 2013). This measure rates studies as being at ‘high’, ‘low’ or ‘unclear’ risk of bias according to various domains. Criteria for the rating of each domain were established by consensus within the research team in accordance with the Cochrane guidelines (online Supplementary Table S2). Quality assessments were carried out independently by ZN, JM and JS and then compared and discrepancies were resolved through discussion and consensus.

### Data extraction

Data were extracted from eligible studies by ZN and JM; uncertainties were resolved by discussion including the third author (JS). The following data were extracted; year of publication; region; duration; interventions; participants' age and inclusion diagnosis; other treatments; previous treatment with lithium; aim of the study and data on suicide and suicidal behaviour, including suicide attempts. In cases of missing data or areas of uncertainty, the corresponding author of the paper was contacted. For trials in which suicide was not reported and a response was not received, it was assumed that no suicides occurred.

### Outcomes and analysis

The primary outcome of interest was suicide. Secondary outcomes were non-fatal suicidal behaviour, as defined by each individual study (this could include suicidal ideation – see Table 1). Due to the heterogeneity of definitions of non-fatal outcomes used in the individual studies, we also decided to look at suicide attempts specifically.

**Table 1.** Characteristics of studies included in this systematic review

Author, year	Region	Duration (weeks)	Participants	Diagnosis	Prior lithium treatment	Aim of intervention	Suicides in lithium group	Suicides in control group	Non-fatal suicidal behaviour in lithium group	Non-fatal suicidal behaviour in control group
Amsterdam, 2010 (Amsterdam and Shults, 2010)	United States	50	Lithium (*N* = 26) Placebo (*N* = 27) Fluoxetine (*N* = 28)	Bipolar II with a current major depressive episode	No participants	Relapse prevention	0	0	0 ‘adverse events’	0 ‘adverse events’
Bauer, 2000 (Bauer *et al*., 2000)	Europe	20	Lithium (*N* = 14) Placebo (*N* = 15)	Major depressive disorder	All participants	Relapse prevention	0	1	Not reported	Not reported
Bowden, 2000 (Bowden *et al*., 2000)	United States	52	Lithium (*N* = 91) Placebo (*N* = 94) Divalproex (*N* = 187)	Bipolar I disorder	Some participants	Relapse prevention	Not reported	Not reported	Not reported	Not reported
Bowden, 2003 (Bowden *et al*., 2003)	Canada, Europe, United States	76	Lithium (*N* = 46) Placebo (*N* = 70) Lamotrigine (*N* = 59)	Bipolar I disorder	Some participants	Relapse prevention	0	0	0 ‘suicide attempts’	0 ‘suicide attempts’
Calabrese, 2003 (Calabrese *et al*., 2003)	Canada, Europe, United States	76	Lithium (*N* = 121) Placebo (*N* = 121) Lamotrigine (*N* = 221)	Bipolar I disorder	Some participants	Relapse prevention	0	0	0 ‘suicide attempts’	1 ‘suicide attempts’
Girlanda, 2014 (Girlanda *et al*., 2014)	Europe	52	Lithium (*N* = 29) Usual care (*N* = 27)	Major depressive disorder	No participants	Suicide prevention	1	0	6 ‘deliberate self-harm’	7 ‘deliberate self-harm’
Katz *et al*., 2022 (Katz *et al*., 2022)	US	52	Lithium (N = 255) Placebo (N = 264)	Bipolar disorder or depression	No participants	Suicide prevention	1^a^	1^a^	65 suicidal behaviour^b^ 16 suicide attempts	62 suicidal behaviour 13 suicide attempts
Lauterbach, 2008 (Lauterbach *et al*., 2008)	Europe	52	Lithium (*N* = 84) Placebo (*N* = 83)	‘Depressive spectrum disorder’	No participants	Suicide prevention	0	3	7 ‘suicide attempts’	7 ‘suicide attempts’
Nierenberg, 2013 (Nierenberg *et al*., 2013)	United States	24	Lithium plus optimalised personalised treatment (OPT) (*N* = 141) OPT alone (*N* = 142)	Bipolar disorder	No participants	Clinical improvement	0	0	Not reported	Not reported
Sackeim, 2001 (Sackeim *et al*., 2001)	United States	24	Lithium (*N* = 28) Placebo (*N* = 29) Nortriptyline (*N* = 27)	Major depressive disorder	Some participants	Relapse prevention	0^a^	0^a^	Not reported	Not reported
Weisler, 2011 (Weisler *et al*., 2011)	Asia, Europe, United States	104	Lithium (*N* = 418) Placebo (*N* = 404) Quetiapine (*N* = 404)	Bipolar I disorder	No participants	Relapse prevention	0	0	3 ‘suicidal behaviour/ ideation’	8 ‘suicidal behaviour/ ideation’
Wilkinson, 2002 (Wilkinson *et al*., 2002)	Europe	104	Lithium (*N* = 25) Placebo (*N* = 24)	Major depressive disorder	No participants	Relapse prevention	0^b^	0^b^	Not reported	Not reported

a Figures for suicides confirmed by authors.

b Suicidal behaviour included: suicide attempts, interrupted suicide attempts and hospitalisation to prevent suicide.

There is no consensus about the methods for meta-analysis of rare events (Efthimiou, 2018). Although the Peto method is the standard procedure in meta-analyses of categorical data (Cochrane Collaboration, 2022), this method may not perform well when the number of trials with zero events is increasing, for unbalanced designs, when the log odds ratio (OR) significantly differs from zero, or when there is no true effect size (Cheng *et al*., 2016; Ren *et al*., 2019). The Peto method may overestimate the treatment effect when there are more trials with zero events in the treatment condition compared to the control condition (Dahabreh and Economopoulos, 2008). Since suicide is rare, and does not occur at all in many trials, the Peto method excludes the majority of available data on lithium and suicide, and may lead to overestimation of the size of a treatment effect (Sharma *et al*., 2017).

Various other methods have been proposed to facilitate analysis of rare and zero events, but most involve approximations, such as continuity corrections, which are not recommended (Efthimiou, 2018) and produce parameters that are difficult to interpret (Lane, 2013). For this reason, it is common to simply pool the data, for example in regulatory assessments of adverse events (Lievre *et al*., 2002; Bradburn *et al*., 2007). We therefore conducted an analysis of the pooled data using the two-sided Fisher's Exact test in R for the comparison of proportions. Following publication of the protocol, we also decided to conduct meta-analysis of ORs, using the Exact method and Bayesian methods, which have been recommended as methods for including trials with zero events (Ren *et al*., 2019). We prioritised the Exact method by Liu *et al*. (2014) over the Bayesian method because the latter may be sensitive to the choice of prior distributions. All analyses were done in R 4.2.0 (R Core Team, 2022). We used the gmeta-package for the Exact method and the rstan/MetaStan packages for the Bayesian analyses. We applied a sceptical, non-informative prior (1/250 < OR < 250) and an informative prior (1/15 < OR < 15). Ninety-five per cent confidence intervals (or credible intervals in the Bayesian analyses) were calculated for each pooled OR, and individual study results and meta-analysis results were displayed in Forest plots. Additionally, we used other meta-analytical methods, including the Peto method, as sensitivity analyses. The R-code is available via the Open Science Framework (https://osf.io/6q3w7/).

For suicide we used data from all trials and assumed that if suicide was not reported then no suicides had occurred. This assumption is reasonable as all trials were published since 2000, after publication of reporting guidance such as the CONSORT statement that specifies the reporting of important harms (Begg *et al*., 1996). As a sensitivity analysis, we looked at trials which explicitly reported suicides or in which we had been able to confirm the occurrence of suicides with the authors. For comparison with previous meta-analyses, we also conducted a sensitivity analysis including trials published before 2000, using data extracted in a previous meta-analysis (Cipriani *et al*., 2005).

Heterogeneity of effect sizes was estimated, if possible, with *I*^2^ and *τ* and the respective 95% confidence intervals. Subgroup analyses were also performed, following Cochrane guidelines to explore heterogeneity between subgroups when estimating treatment effects based on sparse event data (Cochrane Collaboration, 2022). We performed subgroup analyses involving trials in which lithium was used specifically to prevent suicide; trials that did not include people who were taking lithium prior to randomisation; trials involving people with bipolar disorder; and trials involving people with depression spectrum disorder or mixed diagnostic groups.

We applied the same analysis to the secondary outcomes of non-fatal suicidal behaviour and suicide attempts, but we only included trials which explicitly reported on suicidal behaviour in this analysis, since, unlike suicide, we cannot assume this behaviour would be routinely reported.

## Results

### Sample and trial characteristics

**Fig. 1.**
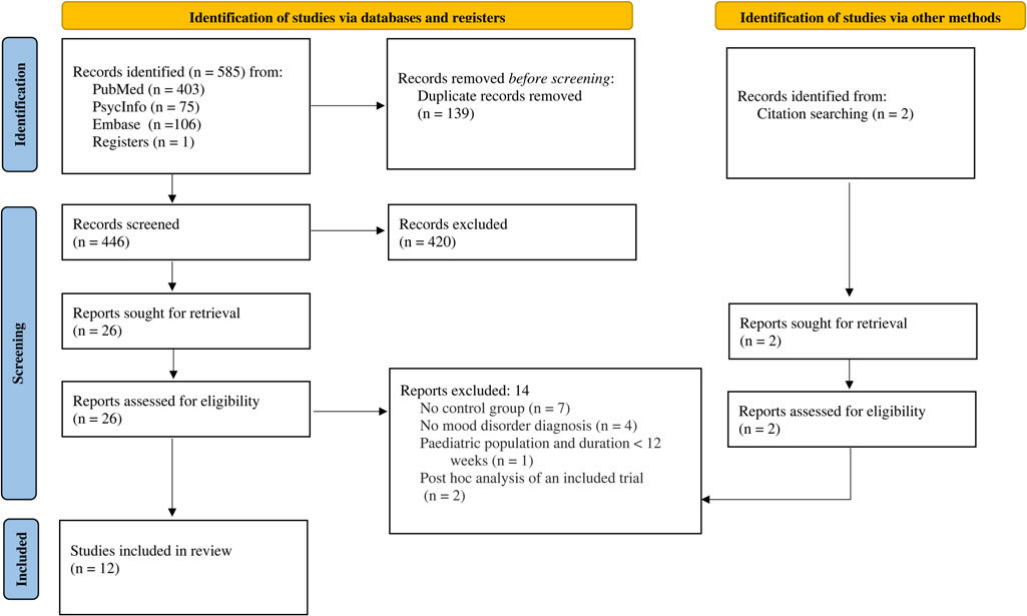
PRISMA flow diagram.

A total of 447 citations were identified after removal of duplicates. After screening, 12 trials published between 2000 and 2021 were identified that satisfied inclusion criteria (see Fig. 1 and online Supplementary Table S3 for details of excluded studies). Table 1 outlines included trial characteristics. Three trials were designed to examine the effect of lithium on suicide and non-fatal suicidal behaviour specifically, and nine trials explored lithium's effect on relapse in people with bipolar disorders or depression. Nine trials reported on suicide explicitly and seven on other suicidal behaviour. The most common comparator was placebo, used in ten trials, and the other two compared lithium with usual care or, in one case, a personalised treatment approach available to all participants (‘optimalised personalised treatment’) (Nierenberg *et al*., 2013). Six trials included participants with bipolar disorder exclusively and six trials included participants with a diagnosis of depression or mixed ‘affective spectrum’ disorders. Follow-up times ranged from 20 to 104 weeks. Six trials included only two arms, while the remaining six trials also compared lithium to another active drug.

### Quality ratings

Risk of bias assessments are displayed in the online Supplementary Fig. S1. Overall, most trials were judged to be of low risk of bias regarding retention (low attrition), whilst weaknesses concerned lack of detail with reporting randomisation procedures and allocation concealment as well as insufficient masking of treatment allocation. In the two trials in which this was tested and reported, between 65 and 68% of participants randomised to take lithium correctly identified their allocation, whilst rates of correct guessing on placebo were no better than chance (Sackeim *et al*., 2001; Katz *et al*., 2022). In three trials in which data could be checked against study protocols, there was no evidence of selective reporting.

### Analysis of suicide

The main analysis involved all 12 trials, which included 1278 participants randomised to lithium and 1300 randomised to placebo or treatment as usual. Two suicides were identified among people randomised to lithium (0.2% of participants across all included studies) and five among those randomised to placebo or treatment as usual (0.4%). The difference was not statistically significant using Fisher's Exact test; OR = 0.41 (0.03–2.49), *p* = 0.45. The difference was also not statistically significant when the analysis was restricted to the 11 trials in which suicide was explicitly reported or confirmed with the authors; 0.2 *v.* 0.4%; OR = 0.41 (0.04–2.49), *p* = 0.45. We did another sensitivity analysis adding in 15 trials published before 2000, mostly in the 1970s, that were included in the review conducted by Cipriani *et al*. in 2005 (Cipriani *et al*., 2005), updated in 2013 (Cipriani *et al*., 2013) and 2017 (Smith and Cipriani, 2017). There were two suicides among 1953 people treated with lithium in total (0.10%) and seven among 1784 who received placebo (0.39%). The difference was also not statistically significant using Fisher's Exact test (*p* = 0.10; OR = 0.26, 95% CI 0.03–1.37) (see online Supplementary Table S4 for details of the pre-2000 trials).

**Fig. 2.**
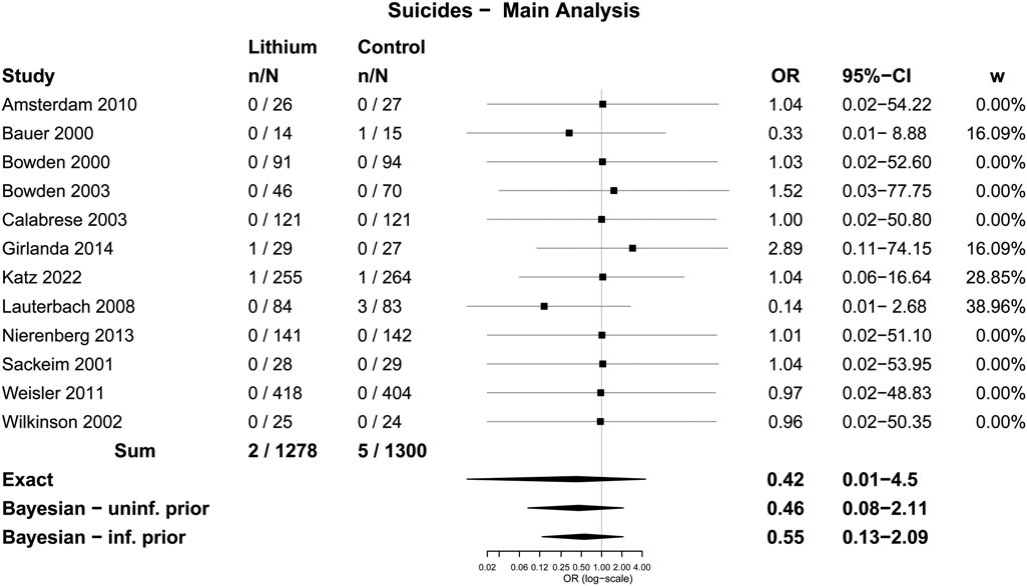
Forest plot: suicides.

**Fig. 3.**
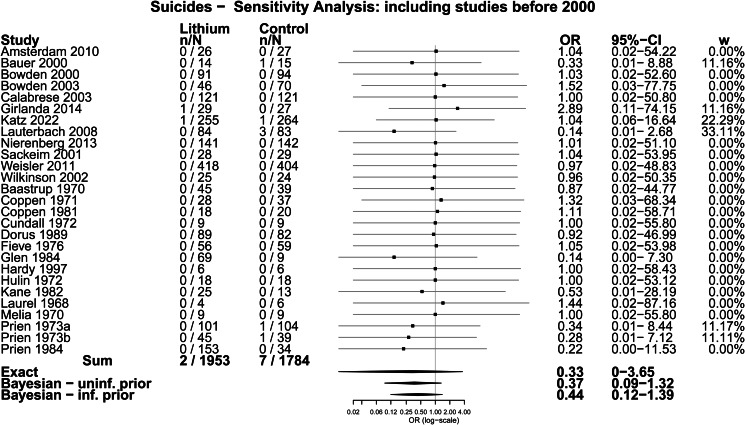
Forest plot: suicides – sensitivity analysis including pre-2000 trials.

Table 2 displays results from the different meta-analytic methods and Fig. 2 shows the associated Forest plot with results from the Exact and Bayesian methods. The OR using the Exact method was 0.42 (0.01–4.5). None of the methods resulted in statistically significant differences between lithium and placebo or treatment as usual. Sensitivity analysis excluding one trial that did not report suicides also did not show a statistically significant difference (online Supplementary Table S5 and Fig. S2). Sensitivity analysis including the 15 trials from before 2000 also did not show statistically significant effects (the Exact method OR was 0.33, 95% CI 0–3.65) (Fig. 3 and online Supplementary Table S6).

**Table 2.** Meta-analysis of suicides

Analysis	Odds ratio (95% CI)	*p*	*I*^2^ (95% CI)	*τ* (95% CI)	*N* (trials)
Exact (Liu *et al*., 2014)	0.42 (0.01–4.5)				12
Baysian^a^ (uninformative prior delta = 250)	0.46 (0.08–2.1)			0.41 (0.01–3.1)	12
Baysian^a^ (informative prior delta = 15)	0.55 (0.13–2.1)			1.41 (1.0–3.1)	12
Non-optimal methods					
Peto (without cc)	0.46 (0.09–2.4)	0.36	18 (0–87)	0.72 (0–6.8)	4
Mantel–Haenszel (without cc)	0.59 (0.13–2.8)	0.51	0 (0–85)	0 (0–4.7)	4
Mantel–Haenszel (with cc)	0.82 (0.29–2.3)	0.70	0 (0–58)	0 (0–0)	12
Mantel–Haenszel (with cc and treatment arm correction)	0.79 (0.28–2.2)	0.65	0 (0–58)	0 (0–0)	12
Arcsine square root transformed risk difference	−0.01 (−0.05 to 0.03)	0.57	0 (–)	0 (–)	12

CI, confidence interval; cc, continuity correction; *N,* number.

a The Bayesian meta-analyses were based on simulations. Because of the special nature of the data (rare events and double zeros), there were occasional large ORs in the posterior after back transforming the log-transformed values of the posterior distribution. The larger ORs skewed the distribution, resulting in slight deviations from sampling to sampling. This affected the upper limits of the credible intervals and the point estimates, but less so the lower limits of the credible intervals. The deviations from sampling to sampling are only minor, affecting mostly the second decimal of the estimations. However, formal analysis provided by the statistical package did not indicate convergence problems. This applies to all the Bayesian analyses performed.

Subgroup analyses involved three trials in which lithium was used specifically to prevent suicide, seven trials involving people who had not taken lithium prior to randomisation, six trials exclusively involving people with bipolar disorders and six trials involving people with depressive disorders or mixed diagnoses. Pooled analysis showed no statistically significant differences in any subgroups (Table 3). Meta-analysis using the Exact method and other methods also showed no statistically significant differences (online Supplementary Tables S7–S10 and Figs S3–S6)

**Table 3.** Pooled subgroup analyses of suicides in randomised control trials comparing lithium and placebo or treatment as usual

	Suicides/*n* (%)				
	Lithium group	Control group	Total	Pooled odds ratio (95% CI)	*p* value (Fisher's Exact test)
Suicide prevention trials (3 trials)	2/368 (0.54%)	4/374 (1.0%)	6/742 (0.81%)	0.51 (0.05–3.6)	0.69
Trials involving people who had not taken lithium prior to randomisation (7 trials)	2/978 (0.20%)	4/971 (0.41%)	6/1949 (0.31%)	0.50 (0.04–3.5)	0.45
Trials exclusively involving people with bipolar disorder (6 trials)	0/843 (0%)	0/858 (0%)	0/1701 (0%)	–	–
Trials involving people with depressive disorder or mixed affective diagnoses (6 trials)	2/435 (0.42%)	5/442 (0.98%)	7/877 (0.71%)	0.40 (0.04–2.5)	0.45

### Analysis of non-fatal suicidal behaviour

Seven trials were included in the analysis of suicidal behaviour, which involved a total of 1975 participants. There were 81 people who undertook some form of non-fatal suicidal behaviour among 1278 participants randomised to lithium (6.3%) and 85 among 1300 people randomised to placebo or treatment as usual (6.5%). The difference was not statistically significant using Fisher's Exact test; OR = 0.97 (0.70–1.34), *p* = 0.87. In five trials that specified suicide attempts, 23 out of 532 people randomised to lithium made a suicide attempt (4.3%), and 21 out of 565 randomised to placebo (3.7%), which was not a statistically significant difference; OR = 1.17 (0.61–2.25), *p* = 0.65.

**Fig. 4.**
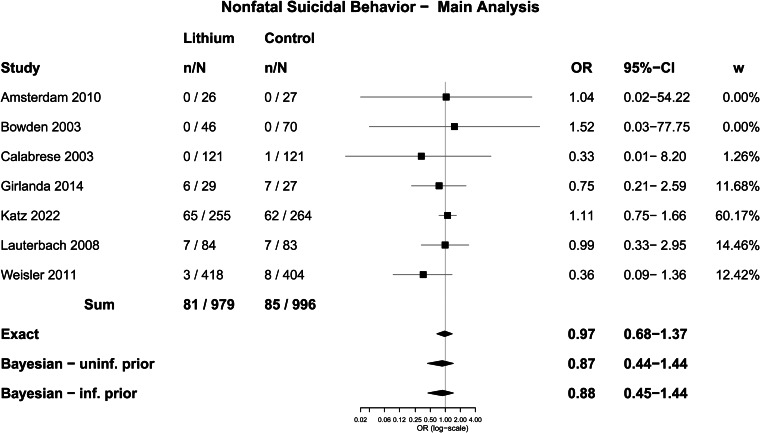
Forest plot: suicidal behaviour.

**Fig. 5.**
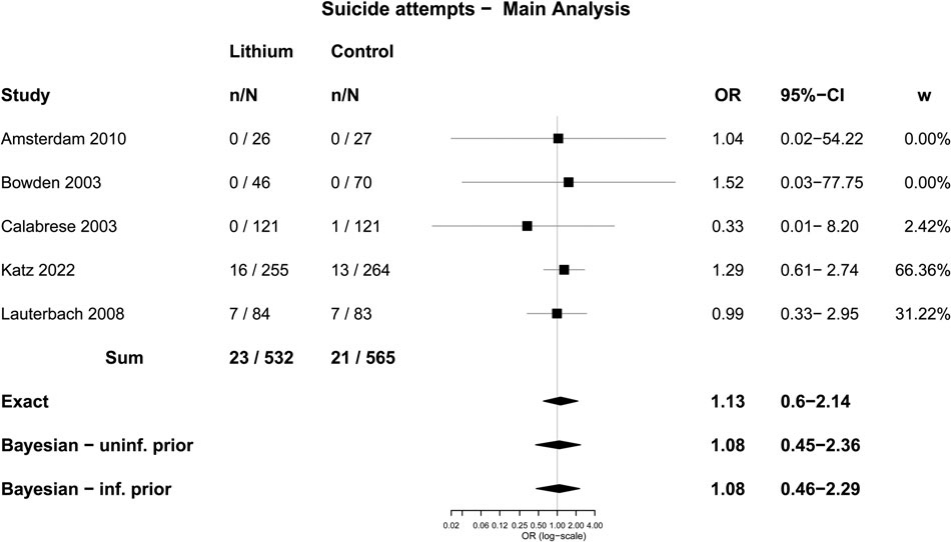
Forest plot: suicide attempts.

Meta-analysis with the Exact method produced an OR of 0.97 (0.68–1.37) for any non-fatal suicidal behaviour, and an OR of 1.13 (0.60–2.14) for suicide attempts. Other methods produced similar results and none showed a statistically significant difference (Table 4, Figs 4 and 5).

**Table 4.** Meta-analysis of suicidal behaviour and suicide attempts

	Suicidal behaviour (7 trials)					Suicide attempts (5 trials)				
Analysis	Odds ratio (95% CI)	*p*	*I*^2^ (95% CI)	*τ* (95% CI)	*N* (trials)	Odds ratio (95% CI)	*p*	*I*^2^ (95% CI)	*τ* (95% CI)	*N* (trials)
Exact (Liu *et al*., 2014)	0.97 (0.68–1.4)				12	1.13 (0.6–2.1)				12
Bayesian (uninformative prior delta = 250)	0.87 (0.44–1.4)			1.31 (1.0–2.4)	12	1.08 (0.45–2.4)			1.3 (1.0–2.7)	12
Bayesian (informative prior delta = 15)	0.88 (0.45–1.4)			1.31 (1.01–2.4)	12	1.08 (0.46–2.3)			1.3 (1.0–2.6)	12
Non-optimal methods										
Peto (without cc)	0.89 (0.57–1.4)	0.61	0 (0–79)	0.21 (0–2)	5	1.12 (0.61–2.1)	0.71	0 (0–90)	0 (0–7.57)	3
Mantel–Haenszel (without cc)	0.94 (0.63–1.4)	0.77	0 (0–79)	0.14 (0–1.4)	5	1.13 (0.62–2.1)	0.69	0 (0–90)	0 (0–4.34)	3
Mantel–Haenszel (with cc)	0.97 (0.68–1.4)	0.86	0 (0–71)	0.07 (0–0.73)	7	1.14 (0.63–2.1)	0.67	0 (0–79)	0 (0–0.81)	5
Mantel–Haenszel (with cc and treatment arm correction)	0.96 (0.66–1.4)	0.82	0 (0–71)	0.1 (0–0.68)	7	1.13 (0.62–2.0)	0.7	0 (0–79)	0 (0–0.71)	5
Arcsine square root transformed risk difference	−0.03 (−0.07 to 0.01)	0.18	0 (–)	0 (–)	7	−0.01 (−0.07 to 0.05)	0.83	0 (–)	0 (–)	5

CI, confidence interval; cc, continuity correction; *N*, number.

## Discussion

Suicide is a complex phenomenon whose epidemiology and causes vary across cultures (Kirmayer, 2022). The WHO recommends a multi-pronged approach to prevention including individual and societal-level interventions (World Health Organisation, 2022). Our analysis shows that the evidence from randomised trials from the new millennium is highly inconclusive and compatible with lithium being associated with an unchanged, decreased or increased risk of suicide. This challenges the results of previous meta-analyses of randomised trials (Cipriani *et al*., 2013; Smith and Cipriani, 2017), guideline recommendations (Veterans Affairs and Department of Defence, (2019) and the longstanding consensus that lithium reduces the risk of suicide. It contrasts with ecological studies that find associations between lithium levels in drinking water and suicide rates, suggesting they may have been influenced by publication bias, which is evident in some reviews (Eyre-Watt *et al*., 2021). In contrast, our results are consistent with a previous systematic review, although this only included a single trial (Borjesson and Gotzsche, 2019), with an earlier Cochrane review published in 2001, which included four trials in total, two of which were included in the meta-analysis (Burgess *et al*., 2001), and with the fact that the largest trial of lithium for suicide prevention, published in 2021, was terminated early due to lack of effect (Katz *et al*., 2022).

The difference between our results and previous meta-analyses that found statistically significant effects (Cipriani *et al*., 2013) or borderline effects (Riblet *et al*., 2017) is partly due to the accumulation of further data, and partly to the inclusion of much data that were previously excluded due to the use of the Peto method that excludes trials with zero events. Traditionally, such trials have been included by applying a continuity correction, but this renders results less accurate and is not recommended (Friedrich *et al*., 2007; Efthimiou, 2018). Pooling of data is one way to address this situation and include accurate data from trials with zero events and is frequently employed by drug regulatory bodies, which are concerned with identifying rare events (Lievre *et al*., 2002; Bradburn *et al*., 2007). The Exact method and Bayesian methods of meta-analysis, which include data from trials with zero events, have also been recommended recently (Ren *et al*., 2019). Thus, our analysis of suicide is based on 12 trials and 2578 participants in total, whereas the previous most influential analysis included only four trials with 485 participants in total (Cipriani *et al*., 2013)

Another reason for the variation between our findings and prior reviews is that we only included trials published after 2000. We took this decision because reporting standards before 2000 were not as rigorous and therefore we cannot be certain that trials published before this date reliably reported adverse events, including suicides, especially since no studies prior to this date were set up to test for suicide prevention effects specifically. In fact, we know that a suicide was not reported in at least one pre-2000 study paper (Glen *et al*., 1984) (among people randomised to amitriptyline) from data obtained subsequently by Cipriani *et al*. (2013). Ioannidis and Lau (49) examined 192 drug trials published between 1967 and 1999 and found that only 39% adequately reported adverse events. Since suicide is rare, the omission of even one or two events may significantly influence the analysis. Following the publication and widespread adoption of the CONSORT statement guidelines in the late 1990s (Begg *et al*., 1996), we can be more confident that trials would reliably report serious adverse events such as suicide. Our sensitivity analysis including trials published before 2000 revealed two further suicides in people allocated to placebo, but there remained no evidence of a statistically significant difference between lithium and the control condition.

Results of previous analyses have also been influenced by three suicides that occurred in the placebo group in one study, the suicide prevention study by Lauterbach *et al*. (2008). This study was reported as being double-blind, but the authors describe how the blind was broken in cases where participants were suspected of non-adherence due to low levels of blood lithium, or when there was a suspected risk of suicide. The former situation is likely to have been common since mean lithium levels were below the intended therapeutic window for the majority of the trial (Lauterbach *et al*., 2008, p. 474). It is plausible, therefore, that the increased monitoring instituted in unblinded participants to maintain adherence or reduce suicide risk might have resulted in fewer suicides in the lithium group. This interpretation is supported by research that shows that greater access to clinical care and closer monitoring can reduce the risk of suicide in clinical and general populations (Tondo *et al*., 2006; Sakinofsky, 2014).

Risk of bias assessments revealed that most included studies had strengths, such as low attrition, and blinding of assessments. Blinding of participants was judged to be insecure across all trials, however, due to the likelihood of the side effects of lithium revealing the identity of medication. There was no evidence of selective reporting, but this was only possible to check in a minority of trials. The risk of bias tool used did not assess aspects of quality that are particularly relevant to rare outcomes, such as sample size and the quality of reporting of adverse events.

We made several protocol changes in order to make the analysis more robust and comprehensive. Thus we searched some trial registers and we added meta-analysis using different methods suggested recently in the statistical literature conducting all these using R instead of Revman. This allowed us to perform a more sophisticated analysis, and we have made the R code publicly available. We also added a sensitivity analysis including trials published before 2000, in order to compare our findings with those of previous reviews. We changed the terminology for describing our secondary outcome from ‘self-harm’ to ‘suicidal behaviour’, since this reflected the language used in most of the included studies and has a clearer relationship to actual suicide because suicide attempts are defined as actions with the intent to die. We also performed an additional analysis of suicide attempts specifically. Another limitation is that full-text screening was not completed independently by two reviewers for the whole sample.

Traditionally, pooling data are criticised because it neglects between-study heterogeneity and estimates may therefore not be sufficiently conservative. However, no statistically significant effect of lithium was found for any of the outcomes we examined using this method, which was consistent with results produced by the different methods of meta-analysis. Heterogeneity of studies could only be estimated with much imprecision. Subgroup analyses did not identify any obvious sources of variation, such as whether the trial included people at high risk of suicide (as in the trials designed to study suicide prevention specifically), the diagnoses of participants and prior treatment with lithium. However, these analyses were based on small numbers of participants and trials and should be interpreted cautiously.

Another review found similar results to our own, but this review not only excluded studies with zero events, but also studies in which participants had been taking lithium prior to study entry. Thus the analysis of suicide was based on a single study (Lauterbach *et al*., 2008), involving 167 participants (Borjesson and Gotzsche, 2019). The justification for excluding studies in which participants were taking lithium prior to randomisation was that all psychoactive drugs produce withdrawal syndromes, which may heighten suicide risk (Baldessarini and Tondo, 2019; Cohen and Recalt, 2019). One empirical study does indicate that suicidal acts were more common in the year following lithium discontinuation than before lithium was started (Baldessarini *et al*., 1999). In addition, lithium discontinuation is associated with an increased risk of relapse of bipolar disorder above baseline (Suppes *et al*., 1991). In our analysis, one of the included studies reported that suicidal behaviour was more common among participants who stopped their randomised treatment prematurely, but this applied to those who were randomised to both lithium and placebo (Katz *et al*., 2022). Our subgroup analysis of trials in which participants had not been treated with lithium previously did not find a difference between lithium and placebo, but the analysis lacked statistical power.

## Conclusion

The evidence from randomised trials of the new millennium is inconclusive and does not provide support for the belief that lithium reduces the risk of suicide or suicidal behaviour. More data are needed to estimate the effect of lithium with more precision in general, and in subgroups of patients, specifically.

## Data Availability

All data are available in the published paper and the Supplementary material. The R code used for meta-analysis is available via the Open Science Framework at https://osf.io/6q3w7/

## Supplementary material

For supplementary material accompanying this paper visit https://doi.org/10.1017/S204579602200049X. click here to view supplementary material

## References

[ref1] Amsterdam JD and Shults J (2010) Efficacy and safety of long-term fluoxetine versus lithium monotherapy of bipolar II disorder: a randomized, double-blind, placebo-substitution study. American Journal of Psychiatry 167, 792–800. 20360317 10.1176/appi.ajp.2009.09020284 PMC2896440

[ref2] Baldessarini RJ and Tondo L (2019) Effects of treatment discontinuation in clinical psychopharmacology. Psychotherapy and Psychosomatics 88, 65–70. 30923289 10.1159/000497334

[ref3] Baldessarini RJ, Tondo L and Hennen J (1999) Effects of lithium treatment and its discontinuation on suicidal behavior in bipolar manic-depressive disorders. The Journal of Clinical Psychiatry 60(suppl. 2), 77–84. 10073392

[ref4] Baldessarini RJ, Tondo L, Davis P, Pompili M, Goodwin FK and Hennen J (2006) Decreased risk of suicides and attempts during long-term lithium treatment: a meta-analytic review. Bipolar Disorders 8, 625–639. 17042835 10.1111/j.1399-5618.2006.00344.x

[ref5] Barraclough B (1972) Suicide prevention, recurrent affective disorder and lithium. British Journal of Psychiatry 121, 391–392. 10.1192/bjp.121.4.391 5077096

[ref6] Bauer M, Bschor T, Kunz D, Berghofer A, Strohle A and Muller-Oerlinghausen B (2000) Double-blind, placebo-controlled trial of the use of lithium to augment antidepressant medication in continuation treatment of unipolar major depression. American Journal of Psychiatry 157, 1429–1435. 10964859 10.1176/appi.ajp.157.9.1429

[ref7] Begg C, Cho M, Eastwood S, Horton R, Moher D, Olkin I, Pitkin R, Rennie D, Schulz KF, Simel D and Stroup DF (1996) Improving the quality of reporting of randomized controlled trials. The CONSORT statement. JAMA 276, 637–639. 8773637 10.1001/jama.276.8.637

[ref8] Borjesson J and Gotzsche PC (2019) Effect of lithium on suicide and mortality in mood disorders: a systematic review. The International Journal of Risk & Safety in Medicine 30, 155–166. 31381531 10.3233/JRS-190058

[ref9] Bowden CL, Calabrese JR, McElroy SL, Gyulai L, Wassef A, Petty F, Pope Jr HG, Chou JC, Keck Jr PE, Rhodes LJ, Swann AC, Hirschfeld RM and Wozniak PJ (2000) A randomized, placebo-controlled 12-month trial of divalproex and lithium in treatment of outpatients with bipolar I disorder. Divalproex Maintenance Study Group. Archives of General Psychiatry 57, 481–489. 10807488 10.1001/archpsyc.57.5.481

[ref10] Bowden CL, Calabrese JR, Sachs G, Yatham LN, Asghar SA, Hompland M, Montgomery P, Earl N, Smoot TM and DeVeaugh-Geiss J (2003) A placebo-controlled 18-month trial of lamotrigine and lithium maintenance treatment in recently manic or hypomanic patients with bipolar I disorder. Archives of General Psychiatry 60, 392–400. 12695317 10.1001/archpsyc.60.4.392

[ref11] Bradburn MJ, Deeks JJ, Berlin JA and Russell Localio A (2007) Much ado about nothing: a comparison of the performance of meta-analytical methods with rare events. Statistics in Medicine 26, 53–77. 16596572 10.1002/sim.2528

[ref12] Burgess S, Geddes J, Hawton K, Townsend E, Jamison K and Goodwin G (2001) Lithium for maintenance treatment of mood disorders. The Cochrane Database of Systematic Reviews 3, CD003013. 10.1002/14651858.CD003013 11687035

[ref13] Calabrese JR, Bowden CL, Sachs G, Yatham LN, Behnke K, Mehtonen OP, Montgomery P, Ascher J, Paska W, Earl N and DeVeaugh-Geiss J (2003) A placebo-controlled 18-month trial of lamotrigine and lithium maintenance treatment in recently depressed patients with bipolar I disorder. The Journal of Clinical Psychiatry 64, 1013–1024. 14628976 10.4088/jcp.v64n0906

[ref14] Chan MK, Bhatti H, Meader N, Stockton S, Evans J, O'Connor RC, Kapur N and Kendall T (2016) Predicting suicide following self-harm: systematic review of risk factors and risk scales. British Journal of Psychiatry 209, 277–283. 10.1192/bjp.bp.115.170050 27340111

[ref15] Cheng J, Pullenayegum E, Marshall JK, Iorio A and Thabane L (2016) Impact of including or excluding both-armed zero-event studies on using standard meta-analysis methods for rare event outcome: a simulation study. BMJ Open 6, e010983. 10.1136/bmjopen-2015-010983 PMC5013416 27531725

[ref16] Cipriani A, Pretty H, Hawton K and Geddes JR (2005) Lithium in the prevention of suicidal behavior and all-cause mortality in patients with mood disorders: a systematic review of randomized trials. American Journal of Psychiatry 162, 1805–1819. 16199826 10.1176/appi.ajp.162.10.1805

[ref17] Cipriani A, Hawton K, Stockton S and Geddes JR (2013) Lithium in the prevention of suicide in mood disorders: updated systematic review and meta-analysis. BMJ 346, f3646. 23814104 10.1136/bmj.f3646

[ref18] Cochrane Collaboration (2022) Analysing Data and Undertaking Meta-Analyses In Training. London, UK: Cochrane Collaboration.

[ref19] Cohen D and Recalt A (2019) Discontinuing psychotropic drugs from participants in randomized controlled trials: a systematic review. Psychotherapy and Psychosomatics 88, 96–104. 30923288 10.1159/000496733

[ref20] Dahabreh IJ and Economopoulos K (2008) Meta-analysis of rare events: an update and sensitivity analysis of cardiovascular events in randomized trials of rosiglitazone. Clinical Trials 5, 116–120. 18375649 10.1177/1740774508090212

[ref21] Daly M (2020) Scientists say lithium should be added to drinking water to prevent suicides. Vice, online: https://www.vice.com/en/article/akzyeb/link-between-lithium-in-drinking-water-suicide-study

[ref22] Del Matto L, Muscas M, Murru A, Verdolini N, Anmella G, Fico G, Corponi F, Carvalho AF, Samalin L, Carpiniello B, Fagiolini A, Vieta E and Pacchiarotti I (2020) Lithium and suicide prevention in mood disorders and in the general population: a systematic review. Neuroscience & Biobehavioral Reviews 116, 142–153. 32561344 10.1016/j.neubiorev.2020.06.017

[ref23] Efthimiou O (2018) Practical guide to the meta-analysis of rare events. Evidence-based Mental Health 21, 72–76. 29650528 10.1136/eb-2018-102911 PMC10270432

[ref24] Eyre-Watt B, Mahendran E, Suetani S, Firth J, Kisely S and Siskind D (2021) The association between lithium in drinking water and neuropsychiatric outcomes: a systematic review and meta-analysis from across 2678 regions containing 113 million people. Australian and New Zealand Journal of Psychiatry 55, 139–152. 33045847 10.1177/0004867420963740

[ref25] Friedrich JO, Adhikari NK and Beyene J (2007) Inclusion of zero total event trials in meta-analyses maintains analytic consistency and incorporates all available data. BMC Medical Research Methodology 7, 5. 17244367 10.1186/1471-2288-7-5 PMC1783664

[ref26] Girlanda F, Cipriani A, Agrimi E, Appino MG, Barichello A, Beneduce R, Bighelli I, Bisoffi G, Bisogno A, Bortolaso P, Boso M, Calandra C, Cascone L, Castellazzi M, Corbascio C, Parise VF, Gardellin F, Gennaro D, Hanife B, Lintas C, Lorusso M, Luca A, Luca M, Luchetta C, Lucii C, Maio F, Marsilio A, Mattei C, Moretti D, Nose M, Occhionero G, Papanti D, Pecile D, Percudani M, Prestia D, Purgato M, Restaino F, Romeo S, Sciarma T, Strizzolo S, Tamborini S, Todarello O, Tozzi F, Ziero S, Zotos S and Barbui C (2014) Effectiveness of lithium in subjects with treatment-resistant depression and suicide risk: results and lessons of an underpowered randomised clinical trial. BMC Research Notes 7, 731. 25326163 10.1186/1756-0500-7-731 PMC4210495

[ref27] Glen AI, Johnson AL and Shepherd M (1984) Continuation therapy with lithium and amitriptyline in unipolar depressive illness: a randomized, double-blind, controlled trial. Psychological Medicine 14, 37–50. 6143340 10.1017/s0033291700003068

[ref28] Hawton K, Casanas ICC, Haw C and Saunders K (2013) Risk factors for suicide in individuals with depression: a systematic review. Journal of Affective Disorders 147, 17–28. 23411024 10.1016/j.jad.2013.01.004

[ref29] Higgins JPT and Green SE (2011) Cochrane Handbook for Systematic Reviews of Interventions. Version 5.1.0. London, England: Cochrane Collaboration.

[ref30] Katz IR, Rogers MP, Lew R, Thwin SS, Doros G, Ahearn E, Ostacher MJ, DeLisi LE, Smith EG, Ringer RJ, Ferguson R, Hoffman B, Kaufman JS, Paik JM, Conrad CH, Holmberg EF, Boney TY, Huang GD, Liang MH and Li+ plus I (2022) Lithium treatment in the prevention of repeat suicide-related outcomes in veterans with major depression or bipolar disorder: a randomized clinical trial. JAMA Psychiatry 79, 24–32. 34787653 10.1001/jamapsychiatry.2021.3170 PMC8600458

[ref31] Kirmayer LJ (2022) Suicide in cultural context: an ecosocial approach. Transcultural Psychiatry 59, 3–12. 35179066 10.1177/13634615221076424

[ref32] Lane PW (2013) Meta-analysis of incidence of rare events. Statistical Methods in Medical Research 22, 117–132. 22218366 10.1177/0962280211432218

[ref33] Lauterbach E, Felber W, Muller-Oerlinghausen B, Ahrens B, Bronisch T, Meyer T, Kilb B, Lewitzka U, Hawellek B, Quante A, Richter K, Broocks A and Hohagen F (2008) Adjunctive lithium treatment in the prevention of suicidal behaviour in depressive disorders: a randomised, placebo-controlled, 1-year trial. Acta Psychiatrica Scandinavica 118, 469–479. 18808400 10.1111/j.1600-0447.2008.01266.x

[ref34] Lewitzka U, Jabs B, Fulle M, Holthoff V, Juckel G, Uhl I, Kittel-Schneider S, Reif A, Reif-Leonhard C, Gruber O, Djawid B, Goodday S, Haussmann R, Pfennig A, Ritter P, Conell J, Severus E and Bauer M (2015a) Does lithium reduce acute suicidal ideation and behavior? A protocol for a randomized, placebo-controlled multicenter trial of lithium plus treatment as usual (TAU) in patients with suicidal major depressive episode. BMC Psychiatry 15, 117. 25986590 10.1186/s12888-015-0499-5 PMC4458032

[ref35] Lewitzka U, Severus E, Bauer R, Ritter P, Muller-Oerlinghausen B and Bauer M (2015b) The suicide prevention effect of lithium: more than 20 years of evidence-a narrative review. International Journal of Bipolar Disorders 3, 32. 26183461 10.1186/s40345-015-0032-2 PMC4504869

[ref36] Lievre M, Cucherat M and Leizorovicz A (2002) Pooling, meta-analysis, and the evaluation of drug safety. Current Controlled Trials in Cardiovascular Medicine 3, 6. 11991807 10.1186/1468-6708-3-6 PMC134478

[ref37] Liu D, Liu RY and Xie M (2014) Exact meta-analysis approach for discrete data and its application to 2 x 2 tables with rare events. Journal of the American Statistical Association 109, 1450–1465. 25620825 10.1080/01621459.2014.946318 PMC4302960

[ref38] Memon A, Rogers I, Fitzsimmons S, Carter B, Strawbridge R, Hidalgo-Mazzei D and Young AH (2020) Association between naturally occurring lithium in drinking water and suicide rates: systematic review and meta-analysis of ecological studies. British Journal of Psychiatry 217, 667–678. 10.1192/bjp.2020.128 32716281

[ref39] National Institute of Mental Health (2022) Suicide. Bethesda, Maryland, US: National Institute of Mental Health.

[ref40] Nierenberg AA, Friedman ES, Bowden CL, Sylvia LG, Thase ME, Ketter T, Ostacher MJ, Leon AC, Reilly-Harrington N, Iosifescu DV, Pencina M, Severe JB and Calabrese JR (2013) Lithium treatment moderate-dose use study (LiTMUS) for bipolar disorder: a randomized comparative effectiveness trial of optimized personalized treatment with and without lithium. American Journal of Psychiatry 170, 102–110. 23288387 10.1176/appi.ajp.2012.12060751

[ref41] Nordentoft M, Mortensen PB and Pedersen CB (2011) Absolute risk of suicide after first hospital contact in mental disorder. Archives of General Psychiatry 68, 1058–1064. 21969462 10.1001/archgenpsychiatry.2011.113

[ref42] O'Connor RC, Wetherall K, Cleare S, Eschle S, Drummond J, Ferguson E, O'Connor DB and O'Carroll RE (2018) Suicide attempts and non-suicidal self-harm: national prevalence study of young adults. BJPsych Open 4, 142–148. 29922479 10.1192/bjo.2018.14 PMC6003254

[ref43] Page MJ, McKenzie JE, Bossuyt PM, Boutron I, Hoffmann TC, Mulrow CD, Shamseer L, Tetzlaff JM, Akl EA, Brennan SE, Chou R, Glanville J, Grimshaw JM, Hrobjartsson A, Lalu MM, Li T, Loder EW, Mayo-Wilson E, McDonald S, McGuinness LA, Stewart LA, Thomas J, Tricco AC, Welch VA, Whiting P and Moher D (2021) The PRISMA 2020 statement: an updated guideline for reporting systematic reviews. BMJ 372, n71. 33782057 10.1136/bmj.n71 PMC8005924

[ref44] R Core Team (2022) A Language and Environment for Statistical Computing. Vienna, Austria: R Foundation for Statistical Computing.

[ref45] Ren Y, Lin L, Lian Q, Zou H and Chu H (2019) Real-world performance of meta-analysis methods for double-zero-event studies with dichotomous outcomes using the Cochrane database of systematic reviews. Journal of General Internal Medicine 34, 960–968. 30887438 10.1007/s11606-019-04925-8 PMC6544742

[ref46] Riblet NBV, Shiner B, Young-Xu Y and Watts BV (2017) Strategies to prevent death by suicide: meta-analysis of randomised controlled trials. British Journal of Psychiatry 210, 396–402. 10.1192/bjp.bp.116.187799 28428338

[ref47] Royal College of Psychiatrists (2010) Self-harm, suicide and risk: helping people who self-harm. Final report of a working group. London.

[ref48] Sackeim HA, Haskett RF, Mulsant BH, Thase ME, Mann JJ, Pettinati HM, Greenberg RM, Crowe RR, Cooper TB and Prudic J (2001) Continuation pharmacotherapy in the prevention of relapse following electroconvulsive therapy: a randomized controlled trial. JAMA 285, 1299–1307. 11255384 10.1001/jama.285.10.1299

[ref49] Sakinofsky I (2014) Preventing suicide among inpatients. Canadian Journal of Psychiatry 59, 131–140. 24881161 10.1177/070674371405900304 PMC4079240

[ref50] Schou M, Juel-Nielsen N, Stromgren E and Voldby H (1954) The treatment of manic psychoses by the administration of lithium salts. Journal of Neurology, Neurosurgery, and Psychiatry 17, 250–260. 13212414 10.1136/jnnp.17.4.250 PMC503195

[ref51] Sharma T, Gotzsche PC and Kuss O (2017) The Yusuf-Peto method was not a robust method for meta-analyses of rare events data from antidepressant trials. Journal of Clinical Epidemiology 91, 129–136. 28802674 10.1016/j.jclinepi.2017.07.006

[ref52] Sivertsen B, Hysing M, Knapstad M, Harvey AG, Reneflot A, Lonning KJ and O'Connor RC (2019) Suicide attempts and non-suicidal self-harm among university students: prevalence study. BJPsych Open 5, e26. 31068238 10.1192/bjo.2019.4 PMC6401540

[ref53] Smith KA and Cipriani A (2017) Lithium and suicide in mood disorders: updated meta-review of the scientific literature. Bipolar Disorders 19, 575–586. 28895269 10.1111/bdi.12543

[ref54] Suppes T, Baldessarini RJ, Faedda GL and Tohen M (1991) Risk of recurrence following discontinuation of lithium treatment in bipolar disorder. Archives Of General Psychiatry 48, 1082–1088. 1845226 10.1001/archpsyc.1991.01810360046007

[ref55] Tondo L, Albert MJ and Baldessarini RJ (2006) Suicide rates in relation to health care access in the United States: an ecological study. Journal of Clinical Psychiatry 67, 517–523. 16669716 10.4088/jcp.v67n0402

[ref56] Veterans Affairs and Department of Defence (2019) Clinical Practice Guideline for the Assessment and Management of Patients at Risk for Suicide. Washington DC: Veterans Affairs and Department of Defence.

[ref57] Weintraub MJ, Van de Loo MM, Gitlin MJ and Miklowitz DJ (2017) Self-harm, affective traits, and psychosocial functioning in adults with depressive and bipolar disorders. Journal of Nervous and Mental Disease 205, 896–899. 29077652 10.1097/NMD.0000000000000744 PMC5679240

[ref58] Weisler RH, Nolen WA, Neijber A, Hellqvist A and Paulsson B and Trial 144 Study, I (2011) Continuation of quetiapine versus switching to placebo or lithium for maintenance treatment of bipolar I disorder (Trial 144: a randomized controlled study). Journal of Clinical Psychiatry 72, 1452–1464. 22054050 10.4088/JCP.11m06878

[ref59] Wilkinson D, Holmes C, Woolford J, Stammers S and North J (2002) Prophylactic therapy with lithium in elderly patients with unipolar major depression. International Journal of Geriatric Psychiatry 17, 619–622. 12112158 10.1002/gps.671

[ref60] World Health Organisation (2021a) Suicide Worldwide in 2019. Geneva, Switzerland: World Health Organisation.

[ref61] World Health Organisation (2021b) Suicide: Key Facts. Geneva: World Health Organisation.

[ref62] World Health Organisation (2022) Suicide Prevention. Geneva: World Health Organisation.

