# Supplementary material for: Effects of lithium on suicide and suicidal behaviour: a systematic review and meta-analysis of randomised trials
Source: Epidemiol Psychiatr Sci. 2022 Sep 16;31:e65. doi: 10.1017/S204579602200049X (PMC9533115; doi:10.1017/S204579602200049X)
Supplement: Supplementary file 1 [file S204579602200049Xsup001.docx]

**Effects of lithium on suicide and suicidal behaviour: an inclusive meta-analysis**

**Supplementary material**

**Supplementary Tables**

**Table 1S: Search terms**

| **Database** | **Search query** | | **Results on 01/03/22** |
| --- | --- | --- | --- |
| **PubMed** | (Lithium) AND (mood disorder* OR affective disorder* OR depress* OR bipolar OR schizoaffective OR personality disorder OR dysthymia OR rapid cycling). Filters: Randomised Control Trial, From 2000 – 3000/12/12 | | 403 |
| **PsycINFO** | #1 | Lithium.m_titl. | 5242 |
|  | #2 | Limit 1 to yr=”2000-Current” | 2230 |
|  | #3 | (Mood disorder* OR affective disorder* OR depress* OR bipolar OR schizoaffective OR personality disorder OR dysthymia OR rapid cycling).m_titl. | 157058 |
|  | #4 | Limit 3 to yr=”2000-Current” | 117967 |
|  | #5 | (Randomised control trial OR rct OR trial or random*).m_titl. | 47837 |
|  | #6 | Limit 5 to yr=”2000-Current” | 41773 |
|  | #7 | 1 and 2 and 3 and 4 and 5 and 6 | 75 |
| **Embase** | #1 | Lithium.m_titl. | 25793 |
|  | #2 | Limit 1 to yr=”2000-Current” | 17627 |
|  | #3 | (Mood disorder* OR affective disorder* OR depress* OR bipolar OR schizoaffective OR personality disorder OR dysthymia OR rapid cycling).m_titl. | 255245 |
|  | #4 | Limit 3 to yr=”2000-Current” | 210327 |
|  | #5 | (Randomised control trial OR rct OR trial or random*).m_titl. | 485634 |
|  | #6 | Limit 5 to yr=”2000-Current” | 433885 |
|  | #7 | 1 and 2 and 3 and 4 and 5 and 6 | 106 |

**Table 2S: Operationalised Criteria for Risk of Bias Assessment**

| **Domains** | **General description** | **Criteria for the current review** |
| --- | --- | --- |
| **Selection bias** | This domain assesses whether the random allocation sequence would have produced comparable groups and whether intervention allocations were adequately concealed during enrolment. |  |
| **Performance bias** | This domain assesses whether all participants and personnel were sufficiently blinded as to which intervention each participant received. | Within this review, we assumed that the side effects associated with lithium would have unblinded the participants, resulting in high risk of bias, unless measures were specified to prevent this. |
| **Detection bias** | This domain assesses whether the outcome assessors were blinded to treatment allocation. |  |
| **Attrition bias** | This domain assesses the completeness of outcome data for each outcome. | An overall attrition rate of over 25% was deemed to be high risk of bias. |
| **Reporting bias** | This domain assesses whether outcomes were selectively reported. | This domain was assessed by comparing the study’s results against the protocol. If the authors did not adhere to the outcomes listed within the protocol the domain was scored as high risk of bias. If there was no protocol available, the domain was scored as ‘unclear risk.’ |
| **Other bias** | This domain assesses whether any other concerns were raised. |  |

**Table 3S: Studies excluded at full text screening stage**

| Study | Reason for exclusion |
| --- | --- |
| AstraZeneca. Efficacy and Safety of Quetiapine Versus Quetiapine Plus Lithium in Bipolar Depression (QUALITY). *ClinicalTrialsgov*, April 17th 2009, 2009. https://clinicaltrials.gov/ct2/show/results/NCT00883493?view=results (accessed 8th March 2022). | No placebo or treatment as usual group |
| Bowden CL, Karayal ON, Schwartz JH, Gundapaneni BK, O'Gorman C. Characterizing relapse prevention in bipolar disorder with adjunctive ziprasidone: clinical and methodological implications. *J Affect Disord* 2013; **144**(1-2): 171-5. | No placebo or treatment as usual group |
| Decloedt EH, Lesosky M, Maartens G, Joska JA. Renal safety of lithium in HIV-infected patients established on tenofovir disoproxil fumarate containing antiretroviral therapy: analysis from a randomized placebo-controlled trial. *AIDS Res Ther* 2017; **14**(1): 6. | Participants did not have a mood disorder |
| Findling RL, Robb A, McNamara NK, et al. Lithium in the Acute Treatment of Bipolar I Disorder: A Double-Blind, Placebo-Controlled Study. *Pediatrics* 2015; **136**(5): 885-94. | Paediatric participants and duration less than 12 weeks |
| Forlenza OV, Radanovic M, Talib LL, Gattaz WF. Clinical and biological effects of long-term lithium treatment in older adults with amnestic mild cognitive impairment: randomised clinical trial. *Br J Psychiatry* 2019; **215**(5): 668-74. | Participants did not have a mood disorder |
| Hollander E, Buchsbaum MS, Haznedar MM, et al. FDG-PET study in pathological gamblers. 1. Lithium increases orbitofrontal, dorsolateral and cingulate metabolism. *Neuropsychobiology* 2008; **58**(1): 37-47. | Participants did not have a mood disorder |
| Missio G, Moreno DH, Demetrio FN, et al. A randomized controlled trial comparing lithium plus valproic acid versus lithium plus carbamazepine in young patients with type 1 bipolar disorder: the LICAVAL study. *Trials* 2019; **20**(1): 608. | No placebo or treatment as usual group |
| Nolen WA, Weisler RH. The association of the effect of lithium in the maintenance treatment of bipolar disorder with lithium plasma levels: a post hoc analysis of a double-blind study comparing switching to lithium or placebo in patients who responded to quetiapine (Trial 144). *Bipolar Disord* 2013; **15**(1): 100-9. | A post hoc subgroup analysis of an included trial (Weisler et al, 2011) |
| Parker G, Ricciardi T, Tavella G, Hadzi-Pavlovic D. A Single-Blind Randomized Comparison of Lithium and Lamotrigine as Maintenance Treatments for Managing Bipolar II Disorder. *J Clin Psychopharmacol* 2021; **41**(4): 381-8. | No placebo or treatment as usual group |
| Patino LR, Klein CC, Strawn JR, et al. A Randomized, Double-Blind, Controlled Trial of Lithium Versus Quetiapine for the Treatment of Acute Mania in Youth with Early Course Bipolar Disorder. *J Child Adolesc Psychopharmacol* 2021; **31**(7): 485-93. | No placebo or treatment as usual group |
| Peselow ED, Clevenger S, IsHak WW. Prophylactic efficacy of lithium, valproic acid, and carbamazepine in the maintenance phase of bipolar disorder: a naturalistic study. *Int Clin Psychopharmacol* 2016; **31**(4): 218-23. | No placebo or treatment as usual group |
| Rombold F, Lauterbach E, Felber W, et al. Adjunctive lithium treatment in the prevention of suicidal behavior in patients with depression and comorbid personality disorders. *IntJPsychiatry ClinPract* 2014; **18**(4): 300-3. | A post hoc analysis of a subgroup in an included trial (Lauterbach et al, 2008) |
| Sacca F, Puorro G, Brunetti A, et al. A randomized controlled pilot trial of lithium in spinocerebellar ataxia type 2. *J Neurol* 2015; **262**(1): 149-53. | Participants did not have a mood disorder |
| Sylvia LG, Reilly-Harrington NA, Leon AC, et al. Methods to limit attrition in longitudinal comparative effectiveness trials: lessons from the Lithium Treatment - Moderate dose Use Study (LiTMUS) for bipolar disorder. *Clin Trials* 2012; **9**(1): 94-101 | No placebo or treatment as usual group |

**Table 4S Trials published before 2000**

| Trial | Participant numbers | Diagnosis | Reported suicides |
| --- | --- | --- | --- |
| Baastrup et al, 1970 | Lithium 45  Placebo 39 | Bipolar & unipolar disorder | 0 |
| Coppen et al, 1971 | Lithium 28  Placebo 37 | Bipolar & unipolar disorder | 0 |
| Coppen et al, 1981 | Lithium 18  Placebo 20 | Depression | 0 |
| Cundall et al, 1972 | Lithium 9 placebo 9 | Bipolar disorder | 0 |
| Dorus et al, 1989 | Lithium 89  Placebo 82 | Alcohol misuse with depression | 0 |
| Fieve et al, 1976 | Lithium 56  Placebo 59 | Bipolar & unipolar disorder | 0 |
| Hardy et al, 1997 | Lithium 6  Placebo 6 | Refractory depression | 0 |
| Hullin et al, 1972 | Lithium 18  Placebo 18 | Bipolar disorder, unipolar disorder, schizoaffective disorder | 0 |
| Glen et al, 1984 | Lithium 69  Placebo 9 | Unipolar depression | 0 |
| Kane et al, 1982 | Lithium 11  Lithium plus imipramine 14  Placebo 13 | Bipolar & recurrent unipolar disorder | 0 |
| Laurell & Ottosson, 1968 | Lithium 4  Placebo 6 | Bipolar & unipolar disorder | 0 |
| Melia, 1970 | Lithium 9  Placebo 9 | Recurrent depression | 0 |
| Prien et al, 1973 | Lithium 101  Placebo 104 | Bipolar disorder | 1, placebo |
| Prien et al, 1973 | Lithium 45  Placebo 39 | Bipolar & unipolar disorder | 1, placebo |
| Prien et al, 1984 | Lithium 45  Lithium plus imipramine 74  Placebo 34 | Bipolar & unipolar disorder | 0 |

References:

**Baastrup, P. C., Poulsen, J. C., Schou, M., Thomsen, K. & Amdisen, A.** (1970). Prophylactic lithium: double blind discontinuation in manic-depressive and recurrent-depressive disorders. *Lancet* **2**, 326-330.

**Coppen, A., Abou-Saleh, M. T., Milln, P., Bailey, J., Metcalfe, M., Burns, B. H. & Armond, A.** (1981). Lithium continuation therapy following electroconvulsive therapy. *Br J Psychiatry* **139**, 284-7.

**Coppen, A., Noguera, R., Bailey, J., Burns, B. H., Swani, M. S., Hare, E. H., Gardner, R. & Maggs, R.** (1971). Prophylactic lithium in affective disorders. Controlled trial. *Lancet* **2**, 275-9.

**Cundall, R. L., Brooks, P. W. & Murray, L. G.** (1972). A controlled evaluation of lithium prophylaxis in affective disorders. *Psychol.Med.* **2**, 308-311.

**Dorus, W., Ostrow, D. G., Anton, R., Cushman, P., Collins, J. F., Schaefer, M., Charles, H. L., Desai, P., Hayashida, M., Malkerneker, U. & et al.** (1989). Lithium treatment of depressed and nondepressed alcoholics. *JAMA* **262**, 1646-52.

**Fieve, R. R., Kumbaraci, T. & Dunner, D. L.** (1976). Lithium prophylaxis of depression in bipolar I, bipolar II, and unipolar patients. *Am J Psychiatry* **133**, 925-9.

**Glen, A. I., Johnson, A. L. & Shepherd, M.** (1984). Continuation therapy with lithium and amitriptyline in unipolar depressive illness: a randomized, double-blind, controlled trial. *Psychol Med* **14**, 37-50.

**Hardy, B. G., Shulman, K. I. & Zucchero, C.** (1997). Gradual discontinuation of lithium augmentation in elderly patients with unipolar depression. *J Clin Psychopharmacol* **17**, 22-6.

**Hullin, R. P., McDonald, R. & Allsopp, M. N.** (1972). Prophylactic lithium in recurrent affective disorders. *Lancet* **1**, 1044-6.

**Kane, J. M., Quitkin, F. M., Rifkin, A., Ramos-Lorenzi, J. R., Nayak, D. D. & Howard, A.** (1982). Lithium carbonate and imipramine in the prophylaxis of unipolar and bipolar II illness: a prospective, placebo-controlled comparison. *Arch.Gen.Psychiatry* **39**, 1065-1069.

**Laurell, B. & Ottosson, J. O.** (1968). Prophylactic lithium? *Lancet* **2**, 1245-6.

**Melia, P. I.** (1970). Prophylactic lithium: a double-blind trial in recurrent affective disorders. *Br J Psychiatry* **116**, 621-4.

**Prien, R. F., Caffey, E. M., Jr. & Klett, C. J.** (1973). Prophylactic efficacy of lithium carbonate in manic-depressive illness. Report of the Veterans Administration and National Institute of Mental Health collaborative study group. *Arch Gen Psychiatry* **28**, 337-41.

**Prien, R. F., Klett, C. J. & Caffey, E. M., Jr.** (1973). Lithium carbonate and imipramine in prevention of affective episodes. A comparison in recurrent affective illness. *Arch Gen Psychiatry* **29**, 420-5.

**Prien, R. F., Kupfer, D. J., Mansky, P. A., Small, J. G., Tuason, V. B., Voss, C. B. & Johnson, W. E.** (1984). Drug therapy in the prevention of recurrences in unipolar and bipolar affective disorders. Report of the NIMH Collaborative Study Group comparing lithium carbonate, imipramine, and a lithium carbonate-imipramine combination. *Arch Gen Psychiatry* **41**, 1096-104.

**Table 5S: Sensitivity analysis: Meta-analysis of 11 trials in which suicides were reported or confirmed by the authors (see also Figure 2S)**

| Analysis | Odds ratio (95% confidence interval) | p | I^2^ (95% CI) | τ (95% CI) | N (trials) |
| --- | --- | --- | --- | --- | --- |
| Exact (Liu et al, 2012) | 0.42 (0.01 - 4.12) |  |  |  | 11 |
| Baysian (uninformative prior delta=250) | 0.46 (0.08 - 2.14) |  |  | 1.41 (1.02 - 3.11 | 11 |
| Baysian (informative prior delta=15) | 0.54 (0.13 - 2.11) |  |  | 1.42 (1.02 - 3.1) | 11 |
| Non-optimal methods |  |  |  |  |  |
| Peto (without cc) | 0.46 (0.09 - 2.43) | 0.36 | 18 (0 - 87) | 0.72 (0 - 6.77) | 4 |
| Mantel-Haenszel (without cc) | 0.6 (0.13 - 2.75) | 0.51 | 0 (0 – 85) | 0 (0 - 4.7) | 4 |
| Mantel-Haenszel (with cc) | 0.8 (0.28 - 2.33) | 0.69 | 0 (0 – 60) | 0 (0 - 0) | 11 |
| Mantel-Haenszel (with cc and treatment arm correction) | 0.78 (0.27 - 2.26) | 0.64 | 0 (0 – 60) | 0 (0 - 0) | 11 |
| Arcsine square root transformed risk difference | -0.01 (-0.05 - 0.03) | 0.56 | 0 ( - ) | 0 ( - ) | 11 |

CI confidence interval

cc continuity correction

N number

**Table 6S: Sensitivity analysis: Meta-analysis including 15 trials published before 2000 (see also Figure 3S)**

| Analysis | Odds ratio (95% confidence interval) | p | I^2^ (95% CI) | τ (95% CI) | N (trials) |
| --- | --- | --- | --- | --- | --- |
| Exact (Liu et al, 2012) | 0.33 (0 - 3.7) |  |  |  | 27 |
| Baysian (uninformative prior delta=250) | 0.37 (0.09 - 1.3) |  |  | 1.39 (1.02 - 2.91) | 27 |
| Baysian (informative prior delta=15) | 0.44 (0.12 - 1.39) |  |  | 1.38 (1.01 - 2.91) | 27 |
| Non-optimal methods |  |  |  |  |  |
| Peto (without cc) | 0.32 (0.09 - 1.2) | 0.09 | 0 (0 - 75) | 0 (0 - 3.62) | 6 |
| Mantel-Haenszel (without cc) | 0.49 (0.14 - 1.73) | 0.37 | 0 (0 - 75) | 0 (0 - 2.15) | 6 |
| Mantel-Haenszel (with cc) | 0.72 (0.35 - 1.47) | 0.27 | 0 (0 - 43) | 0 (0 - 0) | 27 |
| Mantel-Haenszel (with cc and treatment arm correction) | 0.79 (0.38 - 1.64) | 0.53 | 0 (0 - 43) | 0 (0 - 0) | 27 |
| Arcsine square root transformed risk difference | -0.02 (-0.05 - 0.02) | 0.30 | 0 ( - ) | 0 ( - ) | 27 |

CI confidence interval

cc continuity correction

N number

**Table 7S: Subgroup analysis: Meta-analysis including suicide prevention trials (see also Figure 3S)**

| Analysis | Odds ratio (95% confidence interval) | p | I^2^ (95% CI) | τ (95% CI) | N (trials) |
| --- | --- | --- | --- | --- | --- |
| Exact (Liu et al, 2012) | 0.53 (0.04 - 3.7) |  |  |  | 3 |
| Baysian (uninformative prior delta=250) | 0.51 (0.07 - 3.1) |  |  | 1.4 (1.02 - 3.2) | 3 |
| Baysian (informative prior delta=15) | 0.61 (0.12 - 2.9) |  |  | 1.4 (1.02 - 3.2) | 3 |
| Non-optimal methods |  |  |  |  |  |
| Peto (without cc) | 0.64 (0.08 - 5.5) | 0.69 | 40 (0 - 81) | 1.2 (0 - 10) | 3 |
| Mantel-Haenszel (without cc) | 0.7 (0.13 - 3.9) | 0.69 | 0 (0 - 90) | 0 (0 - 9.7) | 3 |
| Mantel-Haenszel (with cc) | 0.7 (0.13 - 3.9) | 0.69 | 0 (0 - 90) | 0 (0 - 9.7) | 3 |
| Mantel-Haenszel (with cc and treatment arm correction) | 0.7 (0.13 – 4.0) | 0.79 | 0 (0 - 90) | 0 (0 - 9.8) | 3 |
| Arcsine square root transformed risk difference | -0.02 (-0.2 - 0.15) | 0.69 | 73.2 ( - ) | 0.02 ( - ) | 3 |

CI confidence interval

cc continuity correction

N number

**Table 8S: Subgroup analysis: Meta-analysis including trials** **involving people who had not taken lithium prior to randomization (see also Figure 3S)**

| Analysis | Odds ratio (95% confidence interval) | p | I^2^ (95% CI) | τ (95% CI) | N (trials) |
| --- | --- | --- | --- | --- | --- |
| Exact (Liu et al, 2012) | 0.46 (0.01 - 4.4) |  |  |  | 7 |
| Baysian (uninformative prior delta=250) | 0.55 (0.08 - 2.9) |  |  | 1.4 (1.0 - 3.2) | 7 |
| Baysian (informative prior delta=15) | 0.62 (0.14 - 2.8) |  |  | 1.4 (1.0 - 3.2) | 7 |
| Non-optimal methods |  |  |  |  |  |
| Peto (without cc) | 0.64 (0.08 - 5.5) | 0.69 | 40 (0 - 81) | 1.2 (0 - 10) | 3 |
| Mantel-Haenszel (without cc) | 0.7 (0.13 - 3.9) | 0.69 | 0 (0 - 90) | 0 (0 - 9.7) | 3 |
| Mantel-Haenszel (with cc) | 0.82 (0.22 – 3.0) | 0.76 | 0 (0 - 71) | 0 (0 - 1.3) | 7 |
| Mantel-Haenszel (with cc and treatment arm correction) | 0.82 (0.22 – 3.0) | 0.76 | 0 (0 - 71) | 0 (0 - 1.3) | 7 |
| Arcsine square root transformed risk difference | -0.01 (-0.07 - 0.04) | 0.64 | 23.4 ( - ) | 0 ( - ) | 7 |

CI confidence interval

cc continuity correction

N number

**Table 9S: Subgroup analysis: Meta-analysis including trials exclusively involving people with bipolar disorder (see also Figure 3S)**

| Analysis | Odds ratio (95% confidence interval) | p | I^2^ (95% CI) | τ (95% CI) | N (trials) |
| --- | --- | --- | --- | --- | --- |
| Exact (Liu et al, 2012) | NA (NA - NA) |  |  |  | 6 |
| Baysian (uninformative prior delta=250) | 1.1 (0.02 – 67.1) |  |  | 1.4 (1.0 - 3.1) | 6 |
| Baysian (informative prior delta=15) | 1.01 (0.09 - 11.4) |  |  | 1.4 (1.0 - 3.0) | 6 |
| Non-optimal methods |  |  |  |  |  |
| Peto (without cc) | NA (NA - NA) | NA | NA (NA - NA) | NA (NA - NA) | 0 |
| Mantel-Haenszel (without cc) | NA (NA - NA) | NA | NA (NA - NA) | NA (NA - NA) | 0 |
| Mantel-Haenszel (with cc) | 1.08 (0.22 - 5.4) | 0.93 | 0 (0 - 75) | 0 (0 - 0) | 6 |
| Mantel-Haenszel (with cc and treatment arm correction) | 1 (0.2 - 5.0) | 1 | 0 (0 - 75) | 0 (0 - 0) | 6 |
| Arcsine square root transformed risk difference | 0 (-0.05 - 0.05) | 1 | 0 ( - ) | 0 ( - ) | 6 |

CI confidence interval

cc continuity correction

N number

**Table 10S: Subgroup analysis: Meta-analysis including trials involving people with depressive disorder or mixed affective diagnoses (see also Figure 3S)**

| Analysis | Odds ratio (95% confidence interval) | p | I^2^ (95% CI) | τ (95% CI) | N (trials) |
| --- | --- | --- | --- | --- | --- |
| Exact (Liu et al, 2012) | 0.49 (0.03 - 3.5) |  |  |  | 6 |
| Baysian (uninformative prior delta=250) | 0.42 (0.07 - 2.1) |  |  | 1.4 (1.0 - 3.2) | 6 |
| Baysian (informative prior delta=15) | 0.51 (0.11 - 2.1) |  |  | 1.4 (1.0 - 3.2) | 6 |
| Non-optimal methods |  |  |  |  |  |
| Peto (without cc) | 0.46 (0.09 - 2.4) | 0.36 | 18 (0 - 87) | 0.72 (0 - 6.8) | 4 |
| Mantel-Haenszel (without cc) | 0.6 (0.13 - 2.8) | 0.51 | 0 (0 - 85) | 0 (0 - 4.7) | 4 |
| Mantel-Haenszel (with cc) | 0.67 (0.18 - 2.6) | 0.56 | 0 (0 - 75) | 0 (0 - 2.1) | 6 |
| Mantel-Haenszel (with cc and treatment arm correction) | 0.67 (0.18 - 2.6) | 0.56 | 0 (0 - 75) | 0 (0 - 2.1) | 6 |
| Arcsine square root transformed risk difference | -0.04 (-0.15 - 0.07) | 0.47 | 45.12 ( - ) | 0.01 ( - ) | 6 |

CI confidence interval

cc continuity correction

N number

| **Supplementary Figures:**  **Figure 1S: Risk of bias assessments** | Random sequence generation (selection bias) | Allocation concealment (selection bias) | Blinding of participants and personnel (performance bias) |  | Blinding of outcome assessment (detection bias) | Incomplete outcome data (attrition bias) | Selective reporting (reporting bias) | Other bias |
| --- | --- | --- | --- | --- | --- | --- | --- | --- |
| Amsterdam (2010) | ? | ? | - |  | + | + | ? | + |
| Bauer (2000) | ? | ? | - |  | + | + | ? | + |
| Bowden (2000) | ? | ? | - |  | + | + | ? | + |
| Bowden (2003) | ? | ? | - |  | + | + | ? | + |
| Calabrese (2003) | ? | ? | - |  | + | + | ? | + |
| Girlanda (2014) | + | + | - |  | + | + | + | + |
| Katz (2021) | + | + | - |  | + | - | + | + |
| Lauterbach (2008) | + | ? | - |  | - | - | ? | - |
| Nierenberg (2013) | + | ? | - |  | + | + | + | + |
| Sackeim (2001) | ? | ? | - |  | + | + | ? | + |
| Weisler (2011) | ? | ? | - |  | + | + | ? | - |
| Wilkinson (2002) | ? | ? | - |  | + | + | ? | + |

| + | Low risk of bias |
| --- | --- |
| ? | Unclear risk of bias |
| - | High risk of bias |

**Figure 2S: Forest plot: Sensitivity analysis of trials in which suicides were reported or confirmed by the authors**

**Figure 3S: Forest plot: Subgroup analysis of suicide prevention trials**

**Figure 4S: Forest plot: Subgroup analysis of trials involving people who had not taken lithium prior to randomization**

**Figure 5S: Forest plot: Subgroup analysis of trials involving people with bipolar disorder**

**Figure 6S: Forest plot: Subgroup analysis of trials involving people with depressive disorder or mixed affective diagnoses**
